# Chitosan-Based Active Packaging for Shrimp Preservation: Development, Functionalization, and Industrial Prospects

**DOI:** 10.3390/foods15061043

**Published:** 2026-03-16

**Authors:** Anand Kumar, Gebremichael Gebremedhin Hailu, Abhirup Mitra, Sadaqat Ali, Sapna Baghel, Zefu Wang, Yang Liu, Wen Xia, Yantao Yin, Shucheng Liu, Shuai Wei

**Affiliations:** 1 College of Food Science and Technology, Guangdong Ocean University, Guangdong Provincial Key Laboratory of Aquatic Product Processing and Safety, Guangdong Province Engineering Laboratory for Marine Biological Products, Guangdong Provincial Engineering Technology Research Center of Seafood, Guangdong Provincial Engineering Technology Research Center of Prefabricated Seafood Processing and Quality Control, Zhanjiang 524088, China; 2 Department of Agriculture, Invertis University, Bareilly 243123, UP, India; 3 Department of Bioscience and Biotechnology, Banasthali University, Jaipur 304022, RJ, India; 4 Collaborative Innovation Centre of Seafood Deep Processing, Dalian Polytechnic University, Dalian 116034, China

**Keywords:** chitosan, shrimp preservation, edible films, shelf life, functionalization, antimicrobial, synergy, composite

## Abstract

The global demand for sustainable and effective food preservation techniques has spurred significant interest in biodegradable packaging materials, with chitosan films emerging as a promising solution for extending the shelf life of highly perishable seafood products such as shrimp. This review systematically summarizes recent advances in the development, characterization, and functional enhancement of chitosan-based films for shrimp. Chitosan, derived from chitin, has inherent antimicrobial, antioxidant, and biodegradable properties, making it an ideal candidate for eco-friendly packaging. The key physicochemical and functional properties of chitosan films, including their mechanical strength, barrier performance, and structural characteristics, are discussed. Functional enhancements, such as the incorporation of natural bioactive compounds (e.g., essential oils and plant extracts) and nanofillers, have been shown to significantly improve the antimicrobial efficacy, oxygen and water vapor barrier properties, and mechanical stability of films. A critical aspect of this progress is the synergistic effect achieved by combining chitosan with other antimicrobials, which broadens the spectrum of activity against various bacterial strains and enhances overall preservation efficacy. Recent studies have demonstrated that functionalized chitosan coatings effectively inhibit microbial growth, retard lipid oxidation, and maintain sensory and nutritional quality during refrigerated storage of shrimp. In addition, this review evaluates current limitations related to large-scale production, cost-effectiveness, and regulatory approval for commercial applications. Overall, chitosan-based preservation systems represent a promising approach for sustainable seafood packaging. Future research may focus on industrial scalability, multifunctional film design, and integration with smart/active packaging technologies.

## 1. Introduction

**Figure 1:**
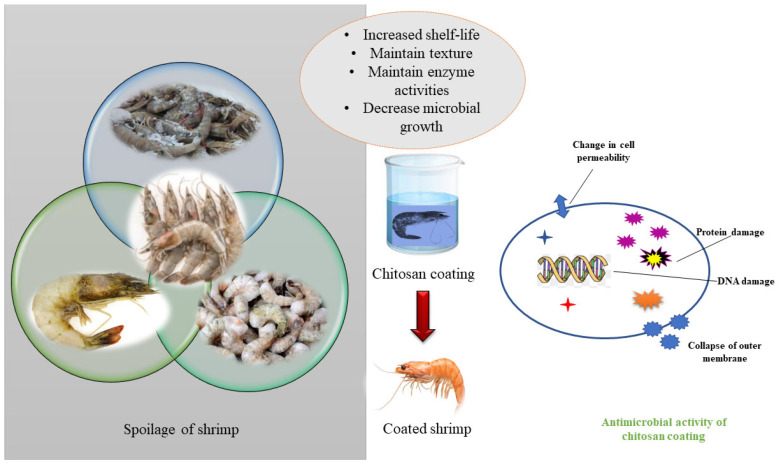
Shrimp spoilage and antimicrobial properties of the chitosan coating.

Plastics have enhanced our day-to-day lives, and our reliance on them has significantly increased in modern living. However, these petrochemicals have their own effects on health and the environment, which is a major concern. Sustainable packaging materials employed for shrimp preservation involve the incorporation of biodegradable materials such as cellulose, starch, and gelatin to form sustainable packaging films. These biodegradable materials are recyclable and easily broken down by the environment. Numerous articles are available demonstrating how various experiments have been conducted to form sustainable packaging alternatives for shrimp preservation; one of which involves the use of bacterial cellulose—produced by *Acetobacter xylinum*—known for its good compatibility, impressive mechanical strength and surface area and is considered a very pure form of cellulose. Bacterial cellulose can support thinner nanofibers, maintaining its beneficial qualities, through a technique called 2,2,6,6-tetramethylpiperidine-1-oxyl (TEMPO) oxidation. For instance, anthocyanin and thymol were introduced to create innovative active packaging materials that could support real-time quality monitoring and add some antioxidant value to packaging material [1]. When chitosan–gelatin composite films were applied to shrimp, bacterial counts were observed to be lower compared to films composed of chitosan alone. The applications of chitosan-based films in the food industry involve antibacterial agents, barrier materials, and sensors, which have made remarkable strides in packaging development [2] (Figure 1).

While this review focuses specifically on shrimp preservation, it is important to recognize that chitosan-based antimicrobial packaging systems demonstrate remarkable versatility across diverse food categories. The fundamental mechanisms by which chitosan films inhibit microbial growth, retard oxidative deterioration, and maintain sensory quality are broadly applicable to various perishable food products. For instance, innovative chitosan composite films have shown exceptional efficacy in preserving fresh produce, meat, poultry, and dairy products, often employing similar functional enhancement strategies such as incorporation of essential oils, plant extracts, and nanoparticles to those discussed for shrimp applications. Notable examples include chitosan coatings enriched with green tea extract and flaxseed oil for beef preservation, which significantly reduced lipid oxidation and extended refrigerated shelf life through synergistic antioxidant and antimicrobial effects [3]. Similarly, chitosan–thyme oil and chitosan–oregano oil composite films have demonstrated enhanced sensory acceptability and microbial control in refreshed meat products [4]. In the poultry sector, chitosan-based films incorporating cinnamon essential oil nanoemulsions effectively suppressed *Salmonella* and *Campylobacter* growth, representing a promising intervention for food safety [5]. Fresh produce preservation has also benefited from chitosan innovations. Chitosan coatings combined with citrus essential oils extended the shelf life of strawberries and grapes by reducing mold growth and delaying senescence [6]. More sophisticated systems, such as chitosan nanoparticles encapsulating curcumin, have been developed for multifunctional food packaging applications, demonstrating broad-spectrum antimicrobial activity and antioxidant properties applicable to both high-protein and high-moisture foods [7].

Chitosan emerges from chitin, which serves as a key element in the cell walls of fungi, exoskeletons of crabs, lobsters, shrimp, and scales of fish and amphibians. Henri Braconnot was credited with the discovery of chitin in 1811, while chitosan was traced back to 1859 through the research of Charles Rougrt, which was later termed by Felix-Hoppe-Seyler in 1894 [8].

The global shrimp industry, which is valued at approximately $57 billion annually, faces persistent challenges in maintaining product quality throughout the supply chain due to the highly perishable nature of crustacean products. Seafood encompasses a variety of marine foods, such as fish, shellfish (crustaceans and mollusks), crabs and other marine animals. The consumption of seafood is experiencing a surge in popularity due to increasing health consciousness and a growing desire for nutritious diet options. In general, seafood is rich in digestible proteins, essential amino acids, astaxanthin, vitamin B12, and polyunsaturated fatty acids, along with other vitamins and minerals such as copper, zinc, sodium, potassium, iodine, and selenium. According to a recent report by the FAO [9], the production of shrimp reached 9.4 million tons in 2022, which represents almost 63% of the increase from the year 2020, specifically in countries such as Vietnam, India, Thailand, China, and Indonesia. However, *Vannamei* shrimp species have been documented to dominate the globe. Postharvest losses in shrimp farming have been a major concern, as shrimps, being perishable in nature, are highly prone to enzymatic oxidation (melanosis); proteolysis, which contributes to microbial attack; and changes in physical, chemical, and organoleptic or sensory properties after shrimp postmortem, which also accounts for postharvest losses in shrimp [10]. Therefore, increasing production and trade in the aquaculture sector in compliance with methods or techniques that could reduce postharvest losses has become important to feed the global population and to mitigate economic concerns in a country [11]. The seafood industry has become one of the most significant contributors to food trading communities, providing a good percentage of the population in developing nations as a source of livelihood and income [12].

However, this sector tends to face challenges due to the perishable nature of seafood. Perishability results from its biological composition, particularly its high-water content of 75–85%. This makes seafood susceptible to microbial and enzymatic activity, as well as lipid oxidation, all of which can lead to sensory deterioration and food safety issues. The resulting enzymatic activity in fish requires water to break down fats and proteins, resulting in texture and flavor changes. Similarly, microbial spoilage and lipid oxidation are also accelerated by the water content present in seafood. Spoilage can be caused at any level of marine food handling, from capture to slaughter, transportation and storage after post-mortem [13]. The practice of transforming marine food into less perishable products is common in developing nations to explore price behavior in fish products [14].

Hence, proper handling and preservation strategies should be adopted to avoid quality losses in seafood, mainly concerning freezing methods, temperature control during the supply chain, and innovative packaging solutions [15]. Priorities should be given to utilizing biodegradable materials and the use of available biomass to reduce spoilage in seafood quality, ensuring sustainability in marine culture [16].

The demand and consumption of high-value marine foods such as shrimp have increased rapidly. China, Thailand, South America, and Asia have emerged as leading players in the seafood trade globally [17]. Shrimp, a popular seafood choice, plays a major role in the global supply chain and is a major export contributing to boosting economic growth for numerous nations. Shrimps are sourced from both wild caught farms and aquaculture farms; however, the wild supply is declining. Two species that are grown substantially are *Litopenaeus vannamei* and *Penaeus monodon*, with the latter stagnating considerably [18].

Combating economy losses due to diseases in shrimp is a major issue influencing global supply and prices, and the prevailing profitability from disease-free shrimp trade has led to geographical relocation [15]. White spot disease caused by white spot syndrome virus has affected the shrimp industry, and antibiotics have been used to combat this disease; however, this has further resulted in the bioaccumulation of harmful and toxic residues in various food webs. Therefore, immunostimulants that can fight infection or disease have been recently introduced to enhance the immune system in a sustainable manner, hence, improving the quality of shrimp, which can derive good economic growth for nations [19].

Seafood, which is highly perishable in nature, needs to be preserved with effective preservation methods and technologies that do not affect the quality of the product and, at the same time, reduce the environmental footprint, ensuring sustainability. This concern has encouraged food industrialists to introduce better and innovative preservation solutions rather than traditional preservation methods for preserving seafood [20]. Non-thermal preservation technologies for seafood preservation involve high-pressure processing (HPP), pulse electric field (PEF), ultrasound, the use of organic acids, natural preservatives, and vacuum packaging [21]. Apart from using traditional and industrial preservation techniques for preserving seafood, packaging methods and materials used for extending the shelf life of any kind of food cannot be ignored.

Diverse applications related to chitosan-based films have been developed in various industries. This review focuses on applications along with the development of chitosan films for extending shrimp shelf life, revealing their sources, properties, functional enhancement, characterization techniques, commercial availability and future perspectives along with sustainability aspects.

### Review Methodology

A systematic literature search was conducted to comprehensively review chitosan-based films for shrimp preservation. The following electronic databases were consulted: Scopus, Web of Science, PubMed, Google Scholar, and ScienceDirect. The search covered the period from 2015 to 2025, capturing recent advances while including foundational studies. The search strategy employed the following Boolean keyword combinations: (chitosan OR chitin) AND (film OR coating OR packaging) AND (shrimp OR prawn OR seafood) AND (preservation OR shelf-life OR spoilage); “chitosan-based film” AND (“antimicrobial activity” OR “antioxidant”) AND “shrimp”; (chitosan AND “plant extract”) OR (chitosan AND “essential oil”) AND “food packaging”; “chitosan characterization” AND (SEM OR FTIR OR XRD OR TGA); “edible film” AND “shrimp”; “biodegradable packaging” AND “seafood”; and “chitosan nanocomposite” AND “barrier properties”. Only articles published in English were considered. The reference lists of selected articles were also manually searched to identify additional relevant studies. The review follows principles of systematic review methodology to ensure comprehensive coverage and reproducibility, though it is not designed as a formal systematic review.

## 2. Chitosan: Sources, Properties, and Roles in Film Development

### 2.1. Sources and Physicochemical Properties of Chitosan

Chitosan is a linear amino-polysaccharide derived from chitin, which serves as a key structural element in the cell walls of fungi and the exoskeletons of crustaceans such as crabs, lobsters, and shrimp. The primary industrial source of chitosan is shrimp processing waste, including shells and exoskeletons discarded during peeling and cooking. This waste stream presents both an environmental challenge and an opportunity for valorization. Shrimp shells consist of chitin (approximately 15–20% by dry weight), proteins (25–40%), minerals (30–50%, mainly ${CaCO}_{3}$), and pigments [22]. Shrimp waste is treated with an alkali–acid solution, which can proximately give around 510 mg/g for chitin and 410 mg/g for chitosan. Demineralization involves the utilization of NaOH and acetic acid. In contrast, organic acids, specifically lactic acid produced by probiotic bacteria for microbial extraction, are employed to demineralize protein extracted from shrimp shells [23]. Chemical extraction remains the main extraction method of chitosan from shrimp shells, and research has focused on determining potential microbial fermentation and green solvent methods for extracting chitin/chitosan [24]. Furthermore, chitosan can also be extracted from non-animal sources such as the discarded stems of mushrooms (e.g., *Agaricus bisporus*), offering a sustainable and animal-free alternative [25].

Since the use of pure chitosan films is limited, due to their poor tensile strength and elasticity, chitin fibers and whiskers are added, creating high-strength biocompatible and biodegradable materials [26]. Chitosan is an amino-polysaccharide; therefore, it is known for its biodegradable and biocompatible properties [27]. Creating chitosan films depends on various processing variables, properties of chitosan, such as its strength, thickness, and types of acids used for dissolving, and the preparation method adopted for making films.

### 2.2. Development of Chitosan-Based Films

The ideal method for developing chitosan-based films varies on the basis of their intended use and the properties needed; for example, for general or simple preparation, the solvent casting method is usually adopted. However, more advanced and specialized applications of these films will require improved qualities with unique structures and different development methods, which might be more suitable. Nevertheless, solvent extraction and solvent casting methods remain the most commonly used methods to make chitosan films. In this process, chitosan is dissolved in an appropriate solvent and then evaporated. The process requires one more step before evaporation, i.e., spreading the solution over a surface to dry, known as casting, which results in a thin chitosan film [28]. Different methods of chitosan film preparation are described in Table 1.

**Figure 2:**
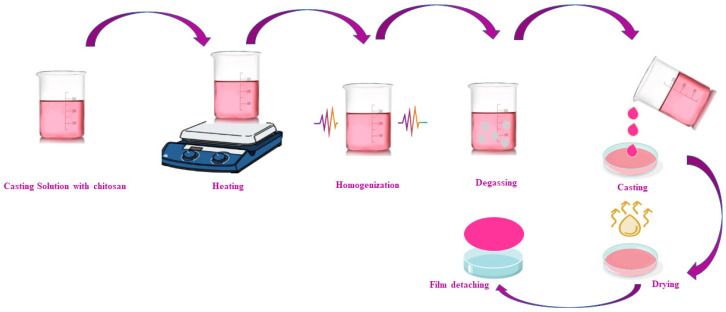
Schematic diagram of chitosan film casting.

However, the resulting films may lack enhanced mechanical properties due to some intermolecular hydrogen bonding; therefore, they are fabricated with various plasticizers such as protein, sugar, and glycerol. The gradual steps involved in this method involve dissolving or blending chitosan with some biopolymers, followed by stirring, filtering and centrifuging the mixture to eliminate air bubbles. Finally, the film is cast or dried and then peeled off [19] (Figure 2).

Constraints regarding the barrier properties of biopolymers have always been a major problem. Therefore, the barrier properties of chitosan films are enhanced by incorporating oil- and water-based antioxidants such as rosemary, ginger, green tea, black tea, and sage in the chitosan matrix [36]. The antimicrobial effectiveness of chitosan can differ, as it is linked to its physical and chemical properties and varies depending on the type of microorganism [37].

Various plasticizers and crosslinkers are incorporated into chitosan films to improve their flexibility and mechanical properties of the film. Glycerol, NaCl and high-pressure homogenization can be used before drying, followed by measuring zeta potential and viscosity of each film. Multilayering of chitosan film is done to enhance its specific properties, such as antimicrobial and antioxidant properties and mechanical and barrier properties. Composite films combine chitosan with other materials such as PVA, gelatin, and sodium alginate. A new method for preparing multilayer composite films has been suggested. This technique involves the step-by-step casting of chitosan and halloysite nanotubes (HNTs) [38].

## 3. Properties, Characterization, and Functional Enhancement of Chitosan-Based Films

### 3.1. Antimicrobial and Antioxidant Properties

The antimicrobial and antioxidant properties of chitosan depend on factors such as its molecular weight, extraction source, and the concentration of chitosan used; in addition, other factors, such as the environmental pH and structural traits of chitosan, affect its antimicrobial properties. Therefore, variations in these characteristics while making chitosan-based films or coatings can significantly affect the permeation of gases through them, as well as their mechanical strength and thermal properties. The preparation of innovative active packaging with chitosan and plant extracts, essential oils, and eco-friendly nanoparticles can further enhance the antimicrobial and antioxidant properties of chitosan.

### 3.2. Physicochemical Properties

Properties such as solubility, molecular weight and degree of deacetylation affect the physicochemical properties of chitosan. Notably, alkaline and neutral solutions cannot dissolve chitosan, but it is soluble in acidic solutions. Additionally, the molecular weight of chitosan is inversely proportional to its solubility, i.e., the greater the molecular weight of the chitosan is, the less soluble it will be. Research has shown that the molecular weight of chitosan depends on the source from which it is extracted. Another important parameter that affects chitosan is its degree of deacetylation. When chitin is converted to chitosan, acetyl groups are removed, resulting in free amino groups; the increased availability of amino groups results in a greater degree of deacetylation (DD), resulting in greater solubility and crystallinity of chitosan [39].

### 3.3. Structural and Crystalline Properties

The structure of chitosan consists of a D-glucosamine backbone connected with glycosidic bonds as well as amino and hydroxyl functional groups. It can show areas of both crystalline and amorphous forms and tends to form complexes with various acids, polymers, and proteins. However, the crystalline structure can be affected by the level of acetylation and the presence of other solvents. The effectiveness of chitosan for specific purposes depends on understanding the physicochemical properties of chitosan.

Chitosan and films made from it are gaining interest among researchers. To improve the biological properties and physical qualities of chitosan-based films, a wide variety of plant extracts and other polymers have been mixed into these films. Specific characteristics of chitosan blends, such as strong mechanical strength and good barrier properties, can only be leveraged for applications in food technology. Despite recent studies on chitosan film production and its usage, research in this area is still limited [40]. Chitosan tends to have limited antibacterial properties as well as very few antioxidant properties; therefore, the properties of chitosan films are enhanced by the introduction of other components, which contribute to the overall properties of chitosan films (Table 2). Combining the natural qualities of chitosan with mechanical enhancements from agricultural waste fillers, these nanofilms significantly improve upon traditional plastic packaging. Natural fillers such as cellulose, starch, and lignin from agricultural byproducts not only strengthen the film but also reduce waste, supporting a circular economy [41]. In a recent report, researchers created curcumin-integrated chitosan nanoparticles and used them to make functional films. Curcumin is recognized as an effective bioactive ingredient for food packaging; as such, it was used in developing a useful nanofiller [7].

Creating films with biopolymers and active ingredients to enhance their functional properties and their effects on the film are synergetic effects. Emulsions and dispersions were created by mixing chitosan–gelatin and pectin–gelatin biopolymers, with lemongrass essential oil or ZnO as the active ingredients. The FTIR spectra of the films indicated that adding glycerol helped form strong hydrogen bonds between glycerol and the chitosan–gelatin or pectin–gelatin composites. This bonding process aids in polymer branching, which is important for encapsulation. Analysis showed that chitosan–gelatin films had lower solubility rates and better mechanical strength than pectin–gelatin films, whereas the presence of lemongrass essential oil enhanced their antibacterial properties. The partial breakdown of the chitosan polymer creates a mixture of polymers and oligomers that can work together to enhance antifungal effects. The combination of chitosan polymer with copper acetate results in an even stronger effect, effectively fighting fungi. The successful development of a new method for synthesizing pure agar–chitosan blends and chitosan–agar/ZnO nanocomposites using in situ chemical synthesis was achieved in a recent study [25].

### 3.4. Analytical and Characterization Techniques

Chitosan films are analyzed through several techniques, including Scanning Electron Microscopy (SEM), to examine their shape, Fourier Transform Infrared Spectroscopy (FTIR), to investigate their chemical structure, X-ray diffraction (XRD), to measure their crystallinity, and Thermogravimetric analysis (TGA). The physical, mechanical, structural and barrier qualities of chitosan films blended with various materials can be analyzed and characterized by these methods. To develop a potential antimicrobial dressing product, researchers merged chitosan with traditional medicinal extracts such as *S. officinalis* and *H. perforatum*, and subsequently examined their effectiveness. They analyzed the structure of this film by SEM, and the chemical structure was analyzed by FTIR. The findings suggest that these chitosan membranes are promising options for wound dressings due to their strong physical and antimicrobial qualities [46]. SEM has revealed that chitosan film morphology directly influences barrier properties and antimicrobial efficacy. Pure chitosan membranes exhibit continuous, compact ridge-and-valley structures free of cracks, explaining their effective barrier against microbial penetration. The incorporation of plant extracts increased surface roughness through the successful integration of bioactive compounds, providing a greater contact area for interaction with microbial cells. Furthermore, FTIR analysis revealed chemical interactions responsible for the enhanced functionality of the films. The increased intensity of O-H stretching bands upon incorporation of the plant extract confirmed hydrogen bonding between chitosan and phenolic compounds, which improves mechanical stability and enables controlled release.

**Figure 3:**
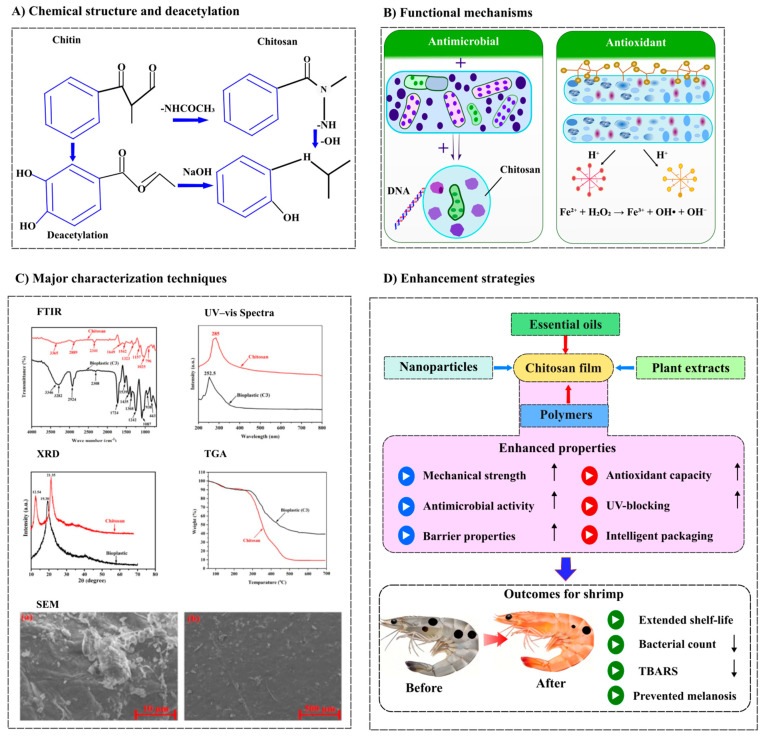
Comprehensive overview of chitosan film functionalization for shrimp preservation. (**A**) Chemical structure: Deacetylation of chitin to chitosan, revealing free amino groups (-${NH}_{2}$) responsible for bioactivity. (**B**) Functional mechanisms: Antimicrobial action via membrane disruption and metal chelation; antioxidant activity via radical scavenging. (**C**) Characterization techniques: SEM (morphology), FTIR (chemical bonds), XRD (crystallinity), and TGA (thermal stability) [49]. (**D**) Enhancement strategies: Incorporation of essential oils, plant extracts, nanoparticles, and polymers improves mechanical, barrier, antimicrobial, and antioxidant properties, extending shrimp shelf life.

Green synthesis is a rapidly growing area of nanobiotechnology, a new biological method that produces chitosan nanoparticles (CNPs) from extracts of *Pelargonium graveolens* leaves. It was optimized using response surface methodology and analyzed using SEM, TEM, FTIR, XRD, TGA and DSC, which confirmed the successful bioconversion process [47]. SEM analysis revealed that chitosan nanoparticles exhibit spherical morphology with uniform size distribution and highly porous surfaces, maximizing surface area for enhanced bacterial binding. XRD analysis confirmed increased amorphous character, with characteristic peaks shifting from $10^{0}$ and $20^{0}$ to $13^{0}$, $19^{0}$, and $35^{0}$, indicating reduced crystallinity that improves sorption properties and biological activity through greater chain mobility and functional group exposure. In addition, High-Performance Liquid Chromatography (HPLC) identified key phenolic compounds in plant extracts incorporated into chitosan membranes. *S. officinalis* contained catechin (11.6 µg/mL), hesperidin (34.7 µg/mL), and rosmarinic acid (29.1 µg/mL), while *H. perforatum* contained epicatechin (90.6 µg/mL), ellagic acid (71.9 µg/mL), and isoquercetin (184.0 µg/mL). These bioactive compounds exert antimicrobial effects through membrane lipid peroxidation and enzyme inhibition, contributing to the broad-spectrum efficacy of functionalized films. Thermal stability analysis confirmed that chitosan nanoparticles retain 20% mass at 800 °C, ensuring film integrity during refrigerated transport. These analytical results clearly indicate that morphological features, chemical interactions, reduced crystallinity, thermal stability, positive surface charge, and bioactive compounds in functionalized chitosan films directly contribute to their superior antimicrobial and barrier properties, enabling extended shrimp shelf life during refrigerated storage [48] (Figure 3).

Even though chitosan films have numerous benefits, their weak mechanical and electrical properties limit their use, which can be enhanced by creating organic–inorganic composites. This can be accomplished by adding fillers such as clays, hydroxyapatite, metal nanoparticles and carbon nanotubes [50]. The physical, mechanical, thermal and functional properties of a chitosan film that uses glycerol as a plasticizer, chitooligosaccharide (COS) as an additive and gallic acid for cross-linking were measured, and the results revealed a much lower moisture content, lower vapor permeability and decreased light transmission as compared to standard chitosan films [45]. Appropriately blended chitosan films were suspended in water, and their physical and chemical properties were assessed, indicating that the water quality remains within acceptable limits. These films also meet strict food-grade standards and were found to help reduce biomass by being incorporated into the natural recycling process [51]. Table 3 summarizes recent studies on chitosan films enhanced with essential oils, plant extracts, and nanoparticles for shrimp preservation.

## 4. Chitosan Film Application in Shrimp Preservation

### 4.1. Application Methods and Effects on Shrimp Quality

**Figure 4:**
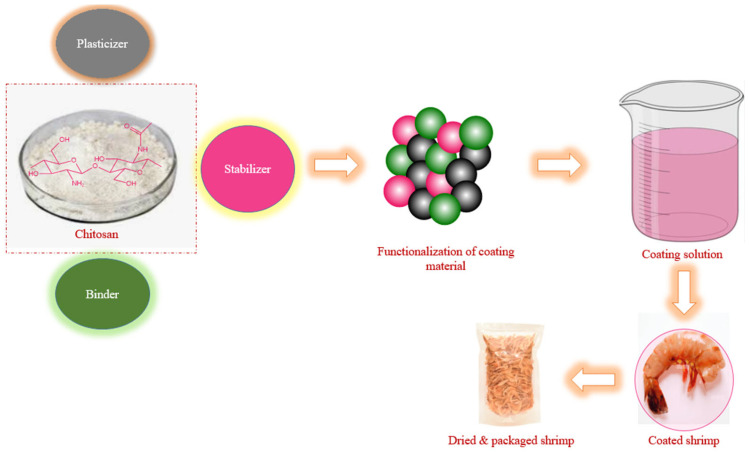
Chitosan coating on shrimp.

Well-blended and fabricated chitosan films can be a promising option for indicating shrimp freshness and slowing down spoilage, as blended chitosan films with other components can change color in response to pH changes, the components of which need to be identified [42] (Figure 4).

**Figure 5:**
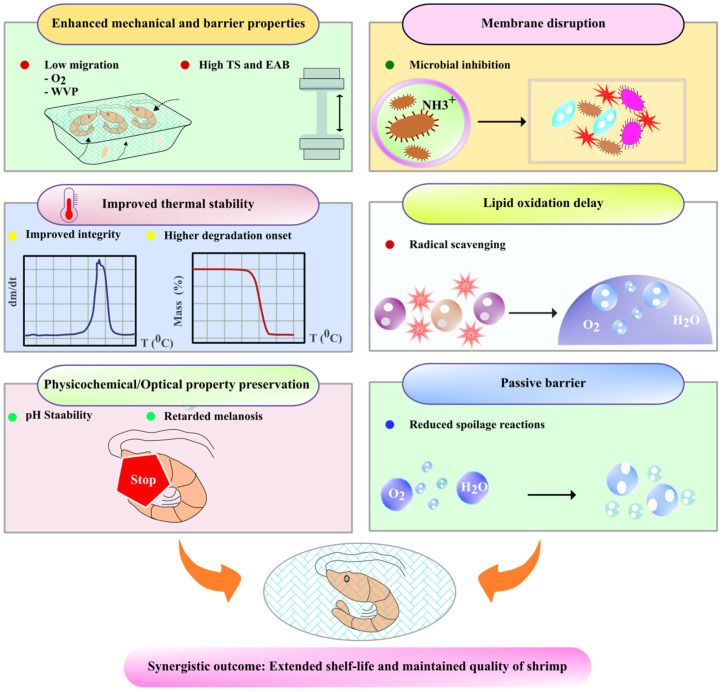
Structure and function of the chitosan-based composite coating for shrimp preservation.

Shrimps can be preserved by application methods such as dipping, spraying or wrapping with chitosan or chitosan films. Water-soluble chitosan can prolong the shelf life of peeled shrimp when stored at cold temperatures (Figure 5). The samples used in this research were analyzed with a 2% chitosan solution and 2% water-soluble chitosan solution, and the results showed that the microbes in the water-soluble chitosan treatment group remained at a minimum until day six, while the pH of the shrimp was 6.8. In conclusion, water-soluble chitosan possesses properties that effectively preserve shrimp and food products [62]. Hence, shrimp can be dipped into or sprayed with a chitosan solution. The bacterial count in the chitosan films was much lower compared to those wrapped in other biopolymer films (here, gelatin) because chitosan has natural antibacterial properties [63].

The left side shows the composite structure of the coating, which integrates chitosan with functional agents (e.g., nanofillers, bioactive compounds) to enhance physical properties such as tensile strength (TS) and elongation at break (EAB). The right side depicts its preservation mechanisms: antimicrobial action (membrane disruption), antioxidant activity (free radical scavenging), and a passive barrier against oxygen and moisture.

Chitosan films affect the physicochemical characteristics of shrimp. Due to differences in chitosan characteristics, the physicochemical properties of shrimp may vary. For example, the concentration of chitosan from pink shrimp shells was recorded to be around 6.9, whereas that of chitosan from sea prawns was 6.7; similarly, the color of chitosan samples from different shrimp sources ranged from white to light yellow. In shrimps, factors such as freezing and heating can also affect their texture, water-holding capacity and weight; studies have found that extrusion methods related to temperature and moisture content have significantly enhanced the protein levels and yields [64]. Current research focuses on examining how ultra-high pressure influences the activities of cathepsins along with the protein oxidation, degradation and quality features of shrimp.

Physicochemical properties, such as Total Volatile Based Nitrogen (TVBN), Thiobarbituric acid reactive substances (TBARS), pH, and microbial plate count, and sensory properties of the shrimp samples should be checked regularly. Researchers grouped shrimp into four categories, i.e., the control group, one with lemon essential oil (LEO), another with basil seed gum (BSG) and the last with BSG + LEO. The TBARS levels, along with the packaging technique adopted, varied among the shrimps during storage, with an appropriate microbial count; comparing control samples showed that the shelf life of the shrimp could be extended periodically [65]. Using hurdle treatments such as salting, drying and irradiation can create effective shelf-stable shrimps that are microbiologically safe and have better sensory qualities [66]. Studies have also shown that coating using tragacanth gum with lime peel extract can extend the shelf life of shrimp, and this combination is recommended for improving shrimp quality during cold storage without any sulfite residue risk [67].

Chitosan films tend to have weak mechanical and barrier properties; therefore, to enhance their physical, chemical and biological characteristics, chitosan was introduced with EAA, i.e., epoxy activated agarose and three types of flavonoids; catechin, quercetin, and luteolin, as the molecular weight of the chitosan increased, the tensile strength (TS), elongation at break (EAB), swelling ability, and thickness of the film also increased. Meanwhile, water vapor permeability (WAP), moisture content, and solubility decrease; hence, the addition of EAA and flavonoids affects the physicochemical and biological properties of chitosan films [68]. Similarly, 25 different chitosan films were formulated with biopolymers for the best performance, such as alginate and glycerol along with cellulose nanocrystals (CNCs) and bacterial nanocellulose (BNC) chitosan films, which presented water vapor transmission rates (WVTR) and oxygen permeability (OTR) decreases; hence, the barrier properties were improved by mixing biopolymers with nanocellulose with chitosan. The addition of cinnamaldehyde (CIN) to chitosan also improved the water resistance of the film [69].

Various studies have indicated that chitosan coatings on shrimp or any other food product have improved their overall quality, color, texture, and odor. Paper packaging using chitosan was developed for roasted coffee beans, and molasses chitosan was also used. The sensory evaluation using Ranking Descriptive Analysis and preference tests revealed that the molasses chitosan coating made the roasted bean darker and that the taste was noticeably different between the chitosan samples; molasses chitosan resulted in higher ratings for overall impression and flavor [70].

The incorporation of essential oils into films not only enhances product shelf life but also contributes to sensory attributes; chitosan–thyme oil and chitosan–oregano oil improve the sensory acceptability of refreshed meat [4]. During shrimp storage, lipid oxidation may cause unacceptable flavors and sensory attributes. Chitosan–gelatin combined with longkong pericarp extract (LPE) was found to reduce PPO enzyme activity, thereby enhancing the quality, taste and shelf life of the product [57].

To improve microbial prevention and retard biochemical changes through chitosan packaging, the focus should be on developing antimicrobial properties either by fabricating them or by other means. Shrimps, as a highly perishable product in nature, are susceptible to microbial attacks and biochemical changes, and studies have proven that using chitosan-fabricated coatings can significantly lower the bacterial count compared with that of shrimp wrapped in other films, such as gelatin films. In a previous study, the bacterial population of shrimp wrapped with chitosan coatings decreased compared with that of the control sample [63]. In biological research, chitosan has been shown to kill *P. gingivalis* bacteria specifically; however, this efficiency is reduced in some delivery systems due to the limited release of peptides [71]. Fresh beef has been preserved with chitosan fabricated with green tea extract, flaxseed oil, vacuum packaging and refrigerated storage [3].

### 4.2. Efficiency in Controlling Microbial and Oxidative Deterioration

To improve microbial prevention and retard biochemical changes through chitosan packaging, enhancing the antimicrobial properties of chitosan is essential. Shrimp are highly perishable and prone to microbial spoilage as well as biochemical deterioration. Research has demonstrated that the application of chitosan-based edible coatings significantly reduces bacterial counts compared to untreated controls or other biopolymer-based films. The efficacy of these systems is often demonstrated through targeted case studies [72,73].

For instance, chitosan films fabricated with suitable materials for specific purposes exhibit antimicrobial and antioxidant properties, affect microbial enzymes and can weaken the microbial cell wall by electrostatic interactions. On the other hand, chitosan, as an antioxidant, prevents oxidative damage by neutralizing free radicals, which contribute to spoilage, and has been proven effective for preserving shrimp, which are susceptible to microbial spoilage and lipid oxidation [31]. In one study, water-soluble chitosan coatings were mixed with Squid Maillard Peptides (SMPs) and stored under chilled conditions and were checked regularly for 20 days. This untreated sample was used as a control, and some samples treated with SMPs and water-soluble chitosan were used to draw a comparison between all the samples [74]. SMPs with chitosan provided the best preservation, at least 16 days, compared to other samples, which lasted for only 8–12 days, due to some beneficial interactions between the water-soluble chitosan and SMPs that reduced microbial growth and controlled lipid oxidation in shrimp during refrigerated storage. The efficacy of various chitosan-based systems in controlling microbial growth, lipid oxidation, and melanosis is further detailed in Table 4.

## 5. Commercial Viability and Challenges

Chitosan composites, either as films or edible coatings, have become periodically significant in the food packaging industry; however, their high cost and reduced thermal and mechanical properties, as well as barrier properties compared to plastic films, limit their widespread use [82]. The production of chitosan is a successful industry, but it faces challenges due to its energy-consuming and wastewater-heavy chemical extraction processes. Many green recovery technologies have opened ways for more sustainable extraction processes, and their adoption is required at larger scale to achieve commercial success [83]. Products made from chitosan tend to stand out as promising alternatives, in terms of mitigation, prevention and control, while addressing environmental problems as well; however, only limited information is available regarding how effectively these materials can perform under adverse conditions and how they can be developed at the industrial scale [84].

Food packaging materials should not alter food taste, odor, or color, which could affect human health or make it unacceptable in any way; in addition, they should be eco-friendly and promote sustainability. Plastics have been widely adopted world-wide for various purposes rather than packaging, due to their ease of commercial availability and low cost, but carry environmental effects; therefore, promising alternative packaging that can serve as a game changer in the packaging industry is needed; therefore, the current focus has shifted toward edible, biodegradable materials that can be converted to appropriate and better packaging materials than plastics [85]. Despite its intrinsic limitations, chitosan has been identified as a promising sustainable packaging material by incorporating various additives, nanoparticles, polymers and other active compounds, thereby increasing its mechanical strength and thermal resistivity as well as enhancing its barrier properties [86]. The task of introducing new compounds into the world is very challenging, and human health safety is the top priority in this case. Commercializing chitosan requires thorough study as well as approval from regulatory bodies such as the FDA in the USA and Codex alimentarius, which seems to be challenging because of the potential impurities, properties and standardized characterization methods of chitosan [87]. The commercial potential of chitosan-based packaging extends across multiple food categories, supporting broader market adoption beyond the seafood sector. Chitosan–cellulose nanocrystal composite films developed for meat packaging exhibit mechanical properties comparable to low-density polyethylene while offering active antimicrobial functionality [87]. These materials have attracted interest from sustainable packaging companies seeking alternatives for fresh meat and poultry applications. Similarly, chitosan-based edible coatings for fruits and vegetables have been commercialized in several Asian markets, where products such as chitocleaner and chitosan are marketed as shelf-life extenders for fresh produce [88].

The cross-sector applicability of chitosan technologies offers significant commercial advantages. Manufacturing infrastructure developed for shrimp packaging films can be adapted for produce, meat, or dairy applications with minimal modification, improving economies of scale and reducing per-unit costs. Companies specializing in biodegradable packaging have begun incorporating chitosan into their product lines in recognition of its versatility across food types. Moreover, successful regulatory approvals for chitosan in one food category, such as the EU’s approval of chitosan as a novel food for certain applications, can streamline approval pathways for related uses [89]. However, challenges specific to different food matrices must be addressed. The high water activity of fresh produce requires different barrier properties compared to fatty fish or meat products, necessitating tailored formulation strategies. Successful commercial scale-up will depend on collaboration among material scientists, food technologists, and packaging engineers to optimize chitosan formulations for specific food categories while maintaining production efficiency. The growing body of research demonstrating chitosan’s efficacy across diverse food types strengthens the business case for investment in large-scale production facilities and regulatory approval processes. Innovative packaging, as an emerging field, is required to meet the demand for environmentally friendly and high-quality packaging, in which chitosan-based packaging seems to have substantial market potential as well as strong innovation potential [70].

Consumer preference changes over time; similarly, consumer preferences have recently shifted towards the quality of food products with extended shelf life and reduced environmental impacts, and preferences have also been assessed by individual sensory criteria through the implementation of the analytical hierarchy process [56]. Society faces food issues related to food nutrition, freshness and food safety, along with low income, to mitigate this issue edible active films or biopolymer films that are biodegradable and tend to enhance overall food quality have been introduced, where chitosan films, especially in the seafood and meat industry, have a considerable impact on this issue, as a major population relies on the meat and seafood industry for their food intake and nutrient requirements [90].

Chitosan films blended with color-changing fluorescent and active features, such as PVA, have the ability to change color, which indicates spoilage and thereby monitors freshness, such as that of pork and shrimp; in addition, adding PVA greatly improved the strength of chitosan films, highlighting their potential as smart and effective options for packaging [79]. The shrimp industry has several negative impacts, such as increased salinity in water bodies, freshwater pollution, high energy requirements and use of fossil fuels, and air pollution. Therefore, adopting green logistic strategies involving planning and managing transportation can lower production costs and improve image and reputation. Hence, it should be noted that an assessment of the life cycle of chitosan, along with its cost-effectiveness, commercial expansion, and properties, is required for its successful replacement with other preservation methods and practices, especially in regard to packaging [82].

## 6. Environmental and Sustainability Aspects

The seafood industry generates enormous amounts of waste in the form of shells, bones, and intestines, which are rich in nutrients; therefore, processing seafood waste becomes important, particularly shrimp waste. There are many methods and approaches for processing sea waste, most of which are harmful; therefore, a safe alternative for bioconversion is required, such as microbial fermentation. Sea waste has a great potential for valorization in various industries such as agrochemicals, therapeutics, and bio-nanomaterials [91]. The global shrimp processing industry produces millions of tons of shell waste annually, and the utilization of this biomass for chitosan production embodies the principles of a circular economy by transforming waste into resources while simultaneously reducing environmental pollution.

Sustainability focused on reusing waste and turning it into valuable products is becoming popular daily, and chitosan products made from molted shrimp shells seem to be a promising source of waste valorization in aquaculture. During shrimp processing, valuable components such as proteins, carotenoids, polysaccharides, and fatty acids are produced. These bioactive compounds have potential applications in various industries due to their antioxidant, anti-inflammatory, and antimicrobial properties. This highlights the importance of sustainable management and a circular economy to increase the value of shrimp waste [92].

From a chemical perspective, the environmental compatibility of chitosan derives fundamentally from its molecular architecture. Chitosan is a linear polysaccharide composed of β-(1→4)-linked D-glucosamine and N-acetyl-D-glucosamine units, containing glycosidic bonds that are susceptible to enzymatic cleavage by naturally occurring microbial enzymes, including lysozyme, chitinase, and chitosanase [93]. The degradation mechanism proceeds through hydrolytic cleavage of polymer chains into smaller oligosaccharides and ultimately monomeric glucosamine units, which undergo complete mineralization to carbon dioxide, water, and biomass through microbial metabolism. Unlike petroleum-based plastics that fragment into persistent microplastics, chitosan undergoes true biodegradation at the molecular level, yielding non-toxic byproducts that can serve as nutrients for soil microbiota and contribute to soil fertility rather than environmental pollution.

Synthetic packaging has been known to contribute to environmental pollution for years. Creating biomaterials such as chitosan can serve as a great substitute for plastic packaging, ensuring sustainability. These films have great potential for fighting bacteria and provide good mechanical properties when they are fabricated with suitable components, and when decomposed, soil microorganisms can use chitosan films for carbon and nitrogen. Chitosan from lobsters is a strong candidate for reducing fossil fuel use and thereby reducing the carbon footprint because of its optical and mechanical properties, which also break down naturally. Continuous assessments are still being conducted based on the life cycle of biodegradable chitosan films and the important factors for the production of chitosan in the future [89].

The environmental benefits of chitosan-based packaging extend beyond shrimp waste valorization to encompass broader sustainability gains across the food supply chain. When applied to multiple food categories, chitosan films contribute to waste reduction at various levels by minimizing food spoilage, replacing petroleum-based plastics, and utilizing seafood processing waste as raw material. Life cycle assessment studies comparing chitosan packaging with conventional plastics for various food applications have demonstrated significant reductions in carbon footprint, particularly when chitosan is derived from waste streams rather than purpose-farmed sources [94].

Chitosan-based packaging, derived from crustacean waste, is a practical model of circular economy principles, transforming a waste stream into a high-value resource. In one major seafood processing hub, this approach has demonstrated substantial benefits. By utilizing shrimp and crab shell waste, the resulting chitosan coating extends fresh produce shelf life by two to three times, inhibits over 80% of common pathogenic bacteria, and reduces moisture loss. This application has the potential to significantly reduce regional fruit and vegetable waste while generating new revenue and mitigating marine waste [95].

**Figure 6:**
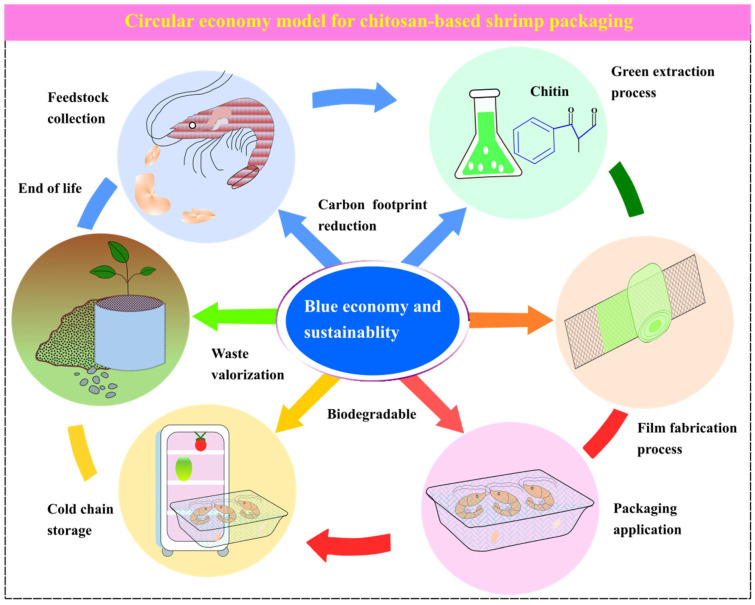
Circular economy model for chitosan-based shrimp packaging. The diagram illustrates the complete life cycle involving feedstock collection from shrimp shell waste, green extraction to obtain chitosan, film fabrication via solvent casting, application to shrimp for shelf-life extension, and end-of-life biodegradation returning nutrients to the environment. The central hub emphasizes waste valorization, reduced fossil fuel dependence, and alignment with circular economy principles.

In a circular economy, waste can be used to enhance the effectiveness of chitosan nanoemulsions and minimize losses of perishable items, contributing to a sustainable economy. These modifications of chitosan, which involve embedding nanoparticles, highlight the importance of blue biotechnology, which indirectly affects the environment and sustainability [96]. Researchers believe that chitosan can be used in sustainable fish farming and to protect the marine environment by turning waste from the seafood industry into valuable nanomaterials, which support new ideas in the blue economy and reduce environmental harm. Since biopolymers such as chitosan have recently replaced synthetic polymers, chemical modifications have become an interesting topic for research to successfully replace them with traditional plastic packaging with limited drawbacks, the main reasons being their nontoxicity and compatibility with environmental protection [97] (Figure 6).

## 7. Future Perspectives

Various antimicrobial agents have been used to fight microbial contamination and spoilage caused by oxidative decay, which poses significant challenges to human health and is a major concern for the food industry. At present, nanoencapsulation is commonly applied for preserving food through antimicrobial systems in the food industry, and nanoencapsulation creates a protective layer that helps shield core materials from harmful reactions and environmental influences [98]. Chitosan is frequently used to encapsulate delicate active ingredients to shield them from breakdown, manage how they are released and enhance their delivery. Due to its natural availability and ability to break down naturally, chitosan is an ideal polymer for encapsulation. Research has shown that chitosan can be used as chitosan-coated nanoliposomes for the encapsulation of caffeine, improving its stability, controlling its release and enhancing its bioavailability [99].

Recently, smart indicators have gained more attention as they detect the quality of food by changing color with dyes that respond to pH levels and are accurate and easy to use; even chitosan has the ability to perform the same function of changing color to indicate the freshness of the food product when linked with dyes such as anthocyanins, suggesting that it is an intelligent freshness indicator [100]. In a previous study, anthocyanin-rich extract from black rice was added to a chitosan composite film, which maintained meat freshness by changing its color from red to blue [81]. Hence, an intelligent system of food packaging offers up-to-date product information to different people involved in the supply chain, such as consumers, retailers and distributors. The future development of chitosan-based packaging will increasingly emphasize cross-category applicability, with innovations in one food sector informing advances in others. Multifunctional films designed for shrimp preservation that incorporate pH-sensitive indicators for freshness monitoring have direct applications in meat and poultry packaging. In these contexts, similar colorimetric responses to spoilage metabolites such as biogenic amines and volatile nitrogen compounds can provide real-time quality information to consumers and retailers [100]. Collaborative research initiatives should focus on identifying common spoilage indicators across food categories, which could inform the design of more versatile chitosan-based packaging systems.

Nanoencapsulation technologies developed for protecting and delivering antimicrobial compounds in shrimp coatings are equally applicable to other food systems. For instance, chitosan-coated nanoliposomes encapsulating essential oils, initially developed for seafood preservation, have been successfully adapted for extending the shelf life of minimally processed fruits and vegetables. In these applications, controlled release of antimicrobial compounds prevents both microbial spoilage and sensory deterioration [101]. Future research should explore the development of platform technologies: chitosan-based delivery systems with modular components that can be customized for specific food matrices while maintaining core functionality. The convergence of intelligent packaging, active compounds, and sustainable materials represents a frontier where innovations for shrimp preservation can catalyze broader food packaging transformations. Cross-disciplinary collaboration between researchers working on different food commodities is essential to identify common challenges and transferable solutions. We encourage future studies to explicitly evaluate the applicability of novel chitosan formulations across multiple food types, generating comparative data that will accelerate technology transfer and commercial adoption. International research networks and multi-commodity research projects could play a valuable role in facilitating this knowledge exchange.

Chitosan films present many opportunities for teamwork and innovation in industry because of their antimicrobial, biodegradable and biocompatible properties. Joint collaborations with university research institutions and businesses can speed up market launch creations and products made from chitosan. Research on chitin/chitosan derived from various sources has focused on increasing its applications in the food, pharmaceutical, and biomedical industries [102]. Considerably, chitosan has also been used in metallic nanoparticles as a biocatalyst, representing its potential as an environmentally friendly alternative [103]. The use of technological features of chitosan also allows the creation of different systems to deliver drugs [104].

## 8. Conclusions

The application of chitosan films for shrimp preservation offers a sustainable and effective alternative to conventional plastic packaging. This review highlights significant progress in enhancing these films through detailed characterization and the incorporation of functional additives. Techniques such as FTIR and SEM provide critical insights into film structure, while the inclusion of natural antimicrobials and nanofillers has markedly improved their barrier properties, mechanical strength, and ability to inhibit microbial growth and lipid oxidation. These advancements are crucial for maintaining shrimp quality and extending shelf life.

The global biodegradable packaging market is projected to experience substantial growth ($700 billion by 2030), with chitosan emerging as a leading biopolymer for active food packaging. Despite its potential, several challenges hinder widespread industrial adoption. Key barriers include high production costs relative to traditional plastics, inherent batch-to-batch variability of the biopolymer, inconsistent regulatory frameworks across regions, and difficulties in scaling up manufacturing processes.

This review is constrained by its non-meta-analytic approach, reliance on English language publications, and the rapid nature of discovery in this field. To overcome existing hurdles, future efforts must focus on optimizing production for cost reduction and consistency. Further research into novel, cost-effective bioactive additives and comprehensive long-term studies on practical, industrial-scale applications are essential. Addressing these gaps will be pivotal for chitosan-based films to revolutionize seafood preservation, enhancing both food security and environmental sustainability.

## Figures and Tables

**Table 1:** Comparison of chitosan film preparation methods for shrimp packaging applications.

Method	Advantages	Limitations	Suitability for Shrimp Preservation	Reference
Solvent casting	Simple, low-cost; blends with PLA/PVA or nano-additives; good oxygen and microbial barrier properties.	Brittle unless plasticized; long drying time; less suitable for large-scale industrial packaging.	PLA/nano-chitosan films extended prawn freshness for up to 18 days under chilled storage.	[29]
	Easy, inexpensive; good antimicrobial barrier; customizable with plasticizers/fillers	Brittle films unless plasticized; long drying times; scale-up challenges	Widely used for shrimp; effective moisture and microbial protection with natural bioactives	[30]
	Simple process; directly applied to shrimp; good barrier and antimicrobial effects	Uniformity issues; thickness variability; may require multiple layers; drying considerations	Well-suited for shrimp; extends shelf life by retarding microbial growth and dehydration	[31]
Electrospinning/electrospray	Produces nanofiber mats with high surface area and antimicrobial effectiveness; improved mechanical strength.	Needs controlled polymer solution properties; complex equipment; limited industrial scale-up so far.	Chitosan/PVA electrospun nanofibers enhance antimicrobial action, promising for active shrimp packaging.	[32]
Layer-by-layer assembly	Precise multilayered coatings; allows for controlled addition of active agents (antioxidants, antimicrobials).	Multiple dipping cycles needed; time-consuming; scalability limitations.	Chitosan/alginate layer-by-layer (LbL) coatings with grapefruit seed extract increased shrimp shelf life by reducing spoilage.	[33]
	Enables multilayer films with tailored functionality; easy incorporation of active agents; biodegradable, antimicrobial properties	Time-consuming; challenging for large-scale production; multiple deposition cycles needed; sensor sensitivity	Promising for controlled-functional active coatings to extend shrimp shelf life	[34]
Chitosan–flavonoid films	Enhanced antioxidant capacity; color indicators for freshness; multifunctional (active + intelligent); dual preservation	Flavonoid release rate must be optimized; color stability may degrade over extended storage.	Flavonoid-loaded chitosan films actively delayed microbial spoilage and indicated shrimp freshness via color change.	[35]

**Table 2:** Mechanical and barrier properties of chitosan films for shrimp packaging.

Film Composition	Tensile Strength (TS)	Elongation at Break (EAB)	Oxygen Permeability (OP)	Water Vapor Permeability (WVP)	Stability & Additional Notes	Reference
Chitosan + Anthocyanin + CNCs (9%)	15 → 35 MPa	Not specified	51.7 → 12.2 g/m^2^·d	31.6 × 10^−12^ → 1.6 × 10^−12^ g/m·s·Pa	Reduced swelling by ~42%; excellent UV barrier, gas & moisture barrier enhanced	[42]
Chitosan + Cellulose/Honey/Curcumin blends	Increased with cellulose	Higher EAB with curcumin blend	Not specified	Improved water resistance; WVP decreased	Improved rigidity (cellulose) or flexibility (curcumin); antioxidant properties enhanced	[43]
Chitosan + ZnO nanoparticles + Gallic acid	Not specified	Not specified	OP reduced by ~41% vs. plain chitosan	WVP decreased up to 56%	Strong antimicrobial and antioxidant effect; improved packaging durability	[44]
Chitosan/PVA/COS + Gallic acid hydrogel	Not specified	Not specified	Not specified	WVP decreased from 14.6 × 10^−9^ to ~7.5 × 10^−9^ g/m·s·Pa	Lower moisture content; improved hydrogen bonding; enhanced barrier performance	[45]

**Table 3:** Functional enhancements of chitosan films with bioactive compounds for the extension of shrimp shelf life.

Additive	Incorporation Method	Antimicrobial Effect	Antioxidant Effect	Impact on Shrimp Quality	Reference
Cinnamon oil nanoemulsion (79 nm)	Film + nanoemulsified cinnamon oil coating	Lower total viable count vs. plain chitosan; effective microbial inhibition over 6 weeks	Reduced TBARS, TVB-N; improved Astaxanthin retention	Delayed lipid oxidation and spoilage; preserved color; antioxidant properties and microbial quality of dried shrimp	[52]
Chitosan nanoparticles + clove extract	Coating with nanochitosan and clove extract	Significant reduction in microbial load during 7 days at 4 °C	High antioxidant activity preserved sensory scores (odor, color, and texture)	Improved microbial safety; treated shrimp remained acceptable for sensory attributes compared to uncoated controls	[53]
Orange peel essential oil (2%)	Solvent-cast chitosan + OPEO film	Delayed microbial spoilage; uncoated shrimp spoiled by day 7, chitosan + OPEO lasted 15 days	Enhanced antioxidant capacity; slowed down oxidation processes	Shrimp coated with chitosan + OPEO film remained acceptable up to 15 days vs. 10 days for plain chitosan and 7 days for control	[54]
Mentha piperita EO (free & nano)	Bilayer carboxymethyl chitosan/pectin + MP EO	Expected reduction in PPO activity and total viable counts during ice storage	Antioxidant action reduces lipid oxidation and melanosis	Targeted control of melanosis (black spot), improved sensory and chemical quality of shrimp during ice storage	[55]
Turmeric essential oil + Silica-magnetic NPs	Solvent-cast chitosan film enriched with TEO-MNP/Si nanoparticles	Controlled bacterial load: ~4.0 → 2.8 log CFU/g over 14 days	Sustained release; delayed oxidation	Extended shelf life and microbial safety of surimi: a model for shrimp preservation	[56]
Chitosan–gelatin + longkong pericarp extract	Coating shrimp with chitosan–gelatin + fruit extract	Inhibited spoilage bacteria (TVB-N suppression)	Polyphenol-rich extract improved antioxidant capacity	Maintained firmness, reduced melanosis, and extended shelf life during chilled storage	[57]
Chitosan + ZnO/TiO_2_/PVA-gelatin NPs	Nanocomposite film applied to white shrimp	Reduced microbial load significantly during ice storage	UV-filtering and free-radical scavenging properties	Enhanced sensory and chemical quality; slowed spoilage in refrigerated shrimp	[58]
Propolis extract fractions	Solvent-cast chitosan film	Significant reduction in bacterial growth (*E. coli*, *S. aureus*)	Elevated DPPH radical scavenging (up to ~70%)	Suggested active packaging; need shrimp-specific studies	[59]
Bio-vanillin + kaolin clay	Film casting: chitosan/KC/BV composite	~90% reduction in *E. coli*, *S. aureus*; ~75% antifungal efficacy	80% antioxidant capacity via DPPH assay	Effective general food packaging; promising model for shrimp	[60]
Purple sweet potato anthocyanin + quercetin-CHI NPs	Intelligent film: CHI nanoparticles with anthocyanin & quercetin	Effective against general foodborne bacteria; used in shrimp freshness monitoring	Strong antioxidant to maintain shrimp color and quality	Designed for shrimp, offers freshness indication alongside shelf-life extension	[35]
Cross-linked chitosan + citric acid + ZnO NPs	Solvent-cast nanocomposite films with ZnO	Zones of inhibition: 16–20 mm against *E. coli*, *P. aeruginosa*, *S. aureus*	Enhanced compared to pure chitosan; improved barrier and radical scavenging	Improved mechanical strength & barrier properties; could handle shrimp packaging demands	[61]

**Table 4:** Efficacy of chitosan-based films and coatings in controlling microbial and oxidative spoilage in shrimp.

Study/Additive	Storage Conditions	Microbial Reduction	Lipid Oxidation (TBARS)	Melanosis Prevention	Reference
Chitosan + Ulva intestinalis nanoliposome	4 °C, ice storage 20 days	Psychrophilic bacteria delayed; TVC lowest in Ch-N-USP treatment	Lower TBA, FFA, PV in Ch-USP and Ch-N-USP samples	PPO inhibited by ~63%, melanosis significantly reduced	[75]
Chitosan + Hyssopus officinalis EO nanoemulsion (1%)	4 °C, 12 days	Psychrophilic: 4.40 ± 0.36 log CFU/g (vs control higher)	TBARS = 0.5 µg/kg (~lowest); significantly less than control	(Not specified)	[76]
Alginate NPs with Z. multiflora + *C. cyminum* EO	4 °C, 15 days	Bacterial count: 2–2.74 log CFU/mL on day 15 (vs. control higher)	TBARS = 1.14 mg MDA/kg (significantly lower than control)	Melanosis score ~2.67 vs. ~8 in control	[77]
Chitosan-gelatin + longkong pericarp extract	4 °C, 20 days	pH changes reduced; implied microbial growth suppression	(Not reported)	Melanosis score ~0–negligible versus increasing in control	[57]
Nanoliposome licorice root + chitosan coating	Ice storage (duration unspecified)	(Not specified)	TBARS ~0.33 mg MDA/kg (stable)	(Not specified)	[75]
Chitosan extracted from prawn shell waste	4 °C ice, 12 days	-	-	Delayed melanosis vs control; control exceeded acceptability by day 8	[78]
Chitosan/anthocyanin/CNC intelligent film	Refrigerated storage (unspecified)	Antibacterial properties	Antioxidant with reduced oxidation	Colorimetric freshness monitoring; delayed spoilage	[79]
Chitosan–PVA/shikonin + ZnO nanoparticles	4 °C & 25 °C, variable time	Strong antibacterial; color shift correlates with TVB-N increases	Effective antioxidant; real-time monitoring	Enables visual indication of spoilage, improving quality control	[80]
Triple-function chitosan/PVA/curcumin-β-CD film	Refrigerated shrimp & pork	Enhanced antibacterial efficacy; ΔE > 5 color change	Improved water barrier, antioxidant properties	Colorimetric and fluorescent indicators of shrimp freshness	[81]

## Data Availability

No new data were created or analyzed in this study. Data sharing is not applicable to this article.
